# Tear deficiency transforms spatial distribution of corneal calcitonin gene-related peptide-positive nerves in rats

**DOI:** 10.3389/fncel.2025.1619310

**Published:** 2025-07-01

**Authors:** Takeshi Kiyoi, Akihiro Nakajima, Qiang He, Li Liu, Shijie Zheng, Shizuka Kobayashi, Junsuke Uwada, Takayoshi Masuoka

**Affiliations:** Department of Pharmacology, School of Medicine, Kanazawa Medical University, Uchinada, Japan

**Keywords:** dry eye, corneal nerve, peptidergic nerves, calcitonin gene-related peptide (CGRP), abnormal sensation, transient receptor potential vanilloid 1 (TRPV1), semaphorin 3A (Sema3A), semaphorin 7A (Sema7A)

## Abstract

The nerve terminals distributed in the cornea are important for sensory perception and the maintenance of ocular surface homeostasis. In dry eye disease (DED), corneal nerves undergo functional and morphological changes that may be involved in abnormal ocular surface sensation and corneal pathology. However, changes in the spatial distribution of corneal nerves, including polymodal nociceptors, and their regulatory mechanisms remain unknown. In the present study, we analyzed time-dependent changes in corneal nerves, focusing on calcitonin gene-related peptide (CGRP)-positive nociceptive nerves in DED model rats, in which both the extraorbital and intraorbital lacrimal glands were surgically excised. After gland excision, the cornea showed acute inflammation, characterized by the presence of segmented-nucleus neutrophil infiltration, followed by chronic inflammation and angiogenesis. In parallel, denervation and subsequent reinnervation in the epithelium, as well as excessive innervation in the stroma, were observed, both involving CGRP-positive nerves. The DED rats showed hypoesthesia and subsequently hyperesthesia in response to mechanical stimulation of the corneal surface, which was synchronized with the denervation and reinnervation of corneal nerve plexuses in the epithelium. Persistent hyperalgesia to capsaicin in DED rats was not correlated with CGRP-positive nerve distribution in the early phase. After gland excision, the expression of neurotropic factor Sema7A increased within the epithelium and stroma, while that of the repulsive axon guidance factor Sema3A decreased in the epithelium. The expression patterns of these molecules correlate with reinnervation of the epithelium and excessive innervation of the stroma. These data suggest that changes in nerve distribution, including CGRP-positive nerves, might partially contribute to sensory perception and progression of corneal inflammatory pathology in DED. Sema3A and Sema7A may be involved in reinnervation as part of the regulatory mechanism in DED.

## 1 Introduction

Dry eye disease (DED) is a major ocular surface disorder characterized by tear film instability, hyperosmolarity, ocular surface inflammation, damage, and neurosensory abnormalities (Nelson et al., 2017). Its onset is influenced by several risk factors including aging, sex, chronic illnesses, use of visual display terminals, and genetic background (Kojima et al., 2011; Uchino et al., 2008). Various subjective symptoms of the ocular surface, such as pain, dryness, and a gritty feeling, appear in patients with DED and significantly reduce their quality of life, which is one of the crucial diagnostic criteria worldwide (Craig et al., 2017; Shimazaki, 2018).

The origin of ocular surface sensation is principally the electrophysiological activity of primary corneal afferents that function as mechano-nociceptors, cold receptors, or polymodal nociceptors. Most calcitonin gene-related peptide (CGRP)-positive sensory nerves are recognized as polymodal nociceptors because they are equipped with transient receptor potential vanilloid 1 (TRPV1) channel detecting noxious heat, acids, and irritants (Belmonte et al., 2017; Guerrero-Moreno et al., 2020). In animal models of dry eye, sensitization of polymodal nociceptors may be involved in hyperalgesia on the ocular surface (Belmonte et al., 2017; Obreja et al., 2002; Sommer and Kress, 2004). One of the sensitization mechanisms is thought to be TRPV1 sensitization, which is mediated by inflammatory cytokines during local inflammation (Immke and Gavva, 2006; Pinho-Ribeiro et al., 2017). However, corneal nerve distribution was also reported to change in DED patients (Labbé et al., 2013) and DED model animals (Fakih et al., 2019; Sullivan et al., 2022); however, the morphological changes in polymodal nociceptive nerve and its relationship with painful sensations on the ocular surface have not been explained in DED. In addition to sensory perception, recent studies have implicated corneal innervation as crucial for maintaining ocular surface homeostasis, such as blinking behavior, tear secretion, immune responses, and epithelial regeneration (Willcox et al., 2017). Thus, the role of abnormal distribution in the corneal nerves is expected to clarify the progression of corneal pathology in DED.

The mechanism underlying the morphological transformation of corneal nerves also remains an unanswered question in DED. Several studies have reported that semaphorin proteins, which have been identified as axon guidance factors, are expressed in the cornea. Semaphorin 3A (Sema3A), a member of the semaphoring family, is categorized as a repulsive axon guidance factor expressed in the epithelium, contributes to proper corneal development by preventing corneal nerve invasion during the early developmental stages (Kubilus and Linsenmayer, 2010). Additionally, corneal epithelial injury changes the expression patterns of Sema3A and its receptor Neuropilin-1, which are associated with wound healing in the epithelium (Guaiquil et al., 2022). The membrane-anchored protein Semaphorin 7A (Sema7A) is known to have an opposite effect to Sema3A, reportedly functioning as a neurotrophic factor and immunomodulatory effector (Pasterkamp et al., 2003); however, expression and function of these semaphorin proteins in cornea during DED have not been investigated.

In this study, we investigated time-dependent changes in the distribution of CGRP-positive corneal nerves, pain-related behaviors, and other corneal pathologies using a severe DED rat model in which both the extraorbital and intraorbital lacrimal glands were excised. Moreover, to reveal the causes of aberrant morphology in corneal nerve fibers, we examined the expression of semaphorin proteins involved in nerve regrowth and remodeling in DED model rats.

## 2 Materials and methods

### 2.1 Animals

This study was approved by the Ethics Committee of the Kanazawa Medical University for Animal Research (approval no. 2023-2). Seven-week-old male Wistar rats were purchased from Japan SLC, Inc (Shizuoka, Japan). The animals were housed at 25 ± 1°C and 55 ± 10% humidity with an automatically controlled 12-h light-dark cycle. Food and water were provided *ad libitum*.

### 2.2 Surgery

**FIGURE 1:**
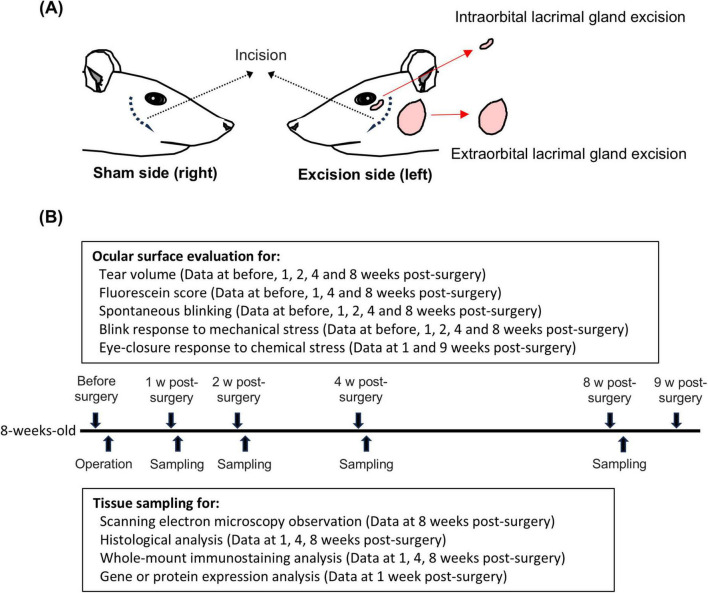
Graphical summary of surgical procedure and experimental timeline. **(A)** Left panel: sham-operated on the right side (Sham). Right panel: extraorbital and intraorbital lacrimal glands surgically excised on left side (Excision). **(B)** Experimental timeline from surgery to 9 weeks post-surgery.

As an animal model of hyposecretory DED, we used 8-week-old rats in which both the extraorbital and intraorbital lacrimal glands were excised on the left side (Excision) and sham-operated on the right side (Sham), as described previously (Meng et al., 2015; Figure 1A). Surgeries were performed under general anesthesia using a combination of intraperitoneal ketamine (90 mg/kg; Daiichi Sankyo, Tokyo, Japan) and xylazine (5 mg/kg; Zenoaq, Fukushima, Japan). At the end of the surgery, the surgical sites were sutured and treated with 0.3% tobramycin (Nitto Medical Co., Toyama, Japan) to prevent infection. The animals were housed in cages for 1–9 weeks after surgery until use.

### 2.3 Experimental schedule

Data on the tearing rate, fluorescein score, number of spontaneous blinks, and positive blink response rate to mechanical stress, described later, were repeatedly acquired from the same animals at each time point from before surgery to 8 weeks after surgery. Subsequently, the tissue samples of these animals were subjected to histopathological and immunohistochemical staining, whole-mount immunostaining, quantitative PCR, and Western blot analyses (Figure 1B). The other animals were exclusively used to measure the eye-closure response induced by chemical stimuli.

### 2.4 Measurement of tear volume

Phenol red-impregnated threads (Zone-Quick; AYUMI Pharmaceutical Co., Tokyo, Japan) were placed under the lower eyelid for 30 s and then removed. The length of the red-stained portion was measured to an accuracy of ± 0.1 mm using a vernier caliper, which reflects the tear volume in the conjunctival sac and tear secretion during the measurement.

### 2.5 Fluorescein staining

Epithelial damage to the bilateral corneas was evaluated by fluorescein staining 1 day before and 1-, 4-, and 8-weeks post-surgery. Fluorescein obtained from the test strip containing 0.7 mg (AYUMI Pharmaceutical Co.) was dissolved with 300 μl of saline (0.9% NaCl). A 2.5 μl of the fluorescein solution was administrated to the ocular surface, followed by washing out with saline. Fluorescence images of the ocular surface were captured using a portable device METORI-50II under blue light (M. E. Technica Inc., Tokyo, Japan). The punctate staining area on the cornea was calculated using ImageJ software (National Institutes of Health, Bethesda, MD, USA) and scored. The scoring criteria were as follows: 0 (stained area, absence of less than one-hundredth of the corneal surface); 1 (stained area, > one-hundredth to less than one-eighth); 2 (stained area, above one-eighth to less than a quarter); 3 (stained area, above one-quarter to less than half); and 4 (stained area, above half).

### 2.6 Scanning electron microscopy observation

More than 24 h after the above experiments, at 8-weeks post-surgery, the rats were decapitated under sufficient isoflurane inhalation and confirmation of respiratory arrest. Both eyeballs were rapidly removed from the decapitated rats. The specimens were incubated in 2% glutaraldehyde in 0.1 M phosphate-buffered saline (PBS; pH 7.4) for 2 h at 4°C and rinsed with 0.1 M PBS (pH 7.4). The samples were stained with 1% osmium tetroxide and 2% tannic acid (OTO processing). After dehydration with ethanol and freeze-drying with t-butyl alcohol, the samples were coated with platinum using an ion coater JFC-1600 (JEOL Ltd., Tokyo, Japan). The corneal surfaces of the samples were observed under a scanning electron microscope (SEM; S3400N; Hitachi High Technologies, Tokyo, Japan).

### 2.7 Measurement of spontaneous blink behavior

Spontaneous blink behavior and tear volume were measured in both eyes 1 day before surgery and 1-, 2-, 4-, and 8-weeks post-surgery. To measure spontaneous blinking behavior, each rat was placed in an acrylic chamber (245 mm × 245 mm × 240 mm) and habituated for 10 min. After habituation, the number of spontaneous blinks was counted for 5 min by two observers. The count was performed twice, averaged, and the average values were recorded. Following the behavioral test, the volume of tear fluid was measured using a phenol red thread test in an unanesthetized state.

### 2.8 Measurement of eye-closure behavior induced by mechanical and chemical stimuli

The center of the cornea in a restrained rat was stimulated with a force of 0.008 *g* using a von Frey filament (514000-20C; Neuro Science, Inc., Tokyo, Japan) to assess the bilateral eye-closure behavior induced by mechanical stimulation 1 day before and 1, 2, 4, and 8 weeks after surgery. Ten stimuli were applied consecutively using filaments and the positive blink response rate was measured. The blink responses were counted separately for those that occurred during filament pressing and immediately after filament release. Each rat was placed in an acrylic chamber and allowed to acclimatize to the chamber for 10 min to assess the eye-closure behavior induced by chemical stimulation in both eyes 1 and 9 weeks after surgery. After the habituation, 5 μl of 100 μM capsaicin in saline was administered to the ocular surface. The series of experiments was recorded using a video camera, and the total eye-closure time within 3 min after the eye drop was measured by two observers watching video images. The data were obtained from different animals at 1 and 9 weeks after surgery.

### 2.9 Histopathological and immunohistochemical staining for paraffine embedded tissue

For histopathological analysis, rats were used 1-, 4-, and 8-weeks postoperatively. The bilateral eyeballs were removed along with the eyelid after transcardial perfusion with 4% paraformaldehyde (PFA) in 0.1 M PBS (pH 7.4) under isoflurane anesthesia. The specimens were then rinsed with 4% PFA in 0.1 M PBS (pH 7.4) for 48 h at 4°C. The fixed specimens were embedded in paraffin and cut sagittally in 4-μm-thickness. The serial 4-μm paraffin-embedded sections were stained with hematoxylin and eosin staining or each immunohistochemical staining. For immunohistochemical staining, the sections were incubated for overnight at 4°C with an anti-tubulin βIII mouse monoclonal antibody (sc-80005; Santa Cruz Biotechnology, Dallas, TX, USA) diluted 1:200, an anti-Sema3A mouse monoclonal antibody (sc-74554, diluted 1:1000; Santa Cruz Biotechnology) and an anti-Sema7A mouse monoclonal antibody (sc-374432, diluted 1:400; Santa Cruz Biotechnology). Subsequently, the sections were treated with either horseradish peroxidase–polymer-labeled anti-mouse immunoglobulin G (IgG) [Histofine Simple Stain rat MAX-PO (M), cat. #414171; Nichirei Biosciences, Tokyo, Japan] or anti-rabbit IgG [Histofine Simple Stain rat MAX-PO (R), cat. #414181; Nichirei Biosciences]. The sections were stained with 3,3’-diaminobenzidine (DAB Substrate Kit, cat. #425011; Nichirei Biosciences). Finally, the slides were counterstained with hematoxylin. Digital images of each slide were captured using a virtual slide system (NanoZoomer C9600; Hamamatsu PhotonicsHamamatsu, Japan). Image analyses were performed using ImageJ software (National Institutes of Health).

For fluorescent immunostaining of Sema7A, the paraffin sections of the corneas at 1- and 4-weeks post-surgery were used and incubated for overnight at 4°C with an anti-Sema7A mouse monoclonal antibody (sc-374432, diluted 1:400; Santa Cruz Biotechnology). Subsequently, the sections were treated with Alexa594-labeled anti-mouse IgG (A11005, diluted 1:400; Thermo Fisher Scientific, Waltham, MA, USA) as a secondary antibody for 1 h at room temperature. Images were acquired using a confocal microscope (LSM710; Zeiss, Oberkochen, Germany).

### 2.10 Whole-mount immunostaining

For whole-mount immunostaining, rats were used at 1-, 4-, and 8-weeks post-operation. After animal decapitation under isoflurane anesthesia as described above, the corneas were rapidly removed and then fixed with periodate-lysine–paraformaldehyde fixative solution for 2 h at 4°C. The specimens were permeabilized with methanol for 1 h at 4°C. The specimens were permeabilized with methanol for 1 h at 4°C. The samples were incubated with Blocking One Histo (06349-64; NACALAI TESQUE, Inc., Kyoto, Japan) for 2 h at room temperature. Thereafter, the specimens were incubated with anti-tubulin βIII mouse monoclonal (sc-80005; Santa Cruz Biotechnology) and anti-CGRP rabbit polyclonal (Y340; Yanaihara Institute Inc., Shizuoka, Japan) as primary antibodies (both diluted 1:200), for 2 days at 4°C. The binding of each antibody was visualized using Alexa488-labeled anti-rabbit IgG (A11008; Thermo Fisher Scientific) and Alexa594-labeled anti-mouse IgG (A11005; Thermo Fisher Scientific) secondary antibodies (both diluted 1:250) for 2 h at room temperature. Images were obtained using a confocal microscope (LSM710; Zeiss).

### 2.11 Quantitative PCR

One week after surgery, the rats were decapitated under isoflurane inhalation and respiratory arrest was confirmed. The trigeminal ganglia were isolated from the excised and sham-operated sides. The total RNA was separately obtained from each whole ganglion using the spin column method (NucleoSpin^®^ RNA Plus, cat. #740984; Macherey-Nagel, Düren, Germany). Real-time PCR was performed using One Step TB Green^®^ PrimeScript™ PLUS RT-PCR Kit (RR096A; Takara Bio Inc., Kusatsu, Japan) and QuantStudio 12 K Flex Real-Time PCR System (Thermo Fisher Scientific). The primers used in this study were: 5′- AGGATGACTACCGGTGGTGTTTC -3′ and 5′ -TCACAGTTGCCTGGGTCCTC -3′ for detection of rat TRPV1 mRNA, 5′- GGCACAGTCAAGGCTGAGAATG -3′ and 5′- ATGGTGGTGAAGACGCCAGTA -3′ for detection of rat glyceraldehyde-3-phosphate dehydrogenase (GAPDH) mRNA. The data were analyzed by the ΔΔCT method with normalizing to expression levels of GAPDH.

### 2.12 Western blot analysis

For western blot analysis, rats were used 1 week post-surgery. Anesthesia and decapitation were performed as previously described. The protein samples were prepared separate from the isolated trigeminal ganglia on the excised and sham sides, separated using electrophoresis, and transferred to polyvinylidene difluoride membranes (033-22453; FUJIFILM Wako Chemical Corporation, Miyazaki, Japan), as previously described (Masuoka et al., 2020a). The membranes were incubated with the appropriate concentrations of anti-VR1 (TRPV1) mouse monoclonal antibody (sc-398417, diluted 1:200; Santa Cruz Biotechnology), anti-phosphate-VR1 (TRPV1) rabbit polyclonal antibody (KM112, diluted 1:500; Trans Genic Inc., Tokyo, Japan), and anti-β-actin (A5316, diluted 1:3000; Sigma -Aldrich, St. Louis, MO, USA) overnight at 4°C. The samples were then treated with a horseradish peroxidase-labeled secondary antibody for mouse IgG (115-035-003, diluted 1:10,000; Jackson ImmunoResearch, West Grove, PA, USA) or anti-rabbit IgG (111-035-003, diluted 1:10,000; Jackson ImmunoResearch) for 2 h at room temperature. Immunoreactive proteins were detected using an EzWestLumi Plus (WSE-7120S; ATTO, Tokyo, Japan). Signal intensity was calculated using ImageJ software (National Institutes of Health).

### 2.13 Statistical analysis

The sample size (*n*) in each experiment was counted as one eye per animal (*n* = 1 per animal), and the data in each group were obtained from different animals. All values are expressed as mean ± SEM. When comparing values between the sham and excision sides, data were analyzed using the Student’s *t*-test, Mann–Whitney U test, or Welch’s *t*-test. Additionally, Bonferroni correction or two-way analysis of variance (ANOVA) was performed as necessary. Statistical significance was set at *P* < 0.05. All statistical analyses were performed using the Sigma Plot Ver. 14.5 software (Systat Software, IL, USA).

## 3 Results

### 3.1 Surgical excision of exorbital and intraorbital lacrimal glands induces tear deficiency and corneal epithelial damage

**FIGURE 2:**
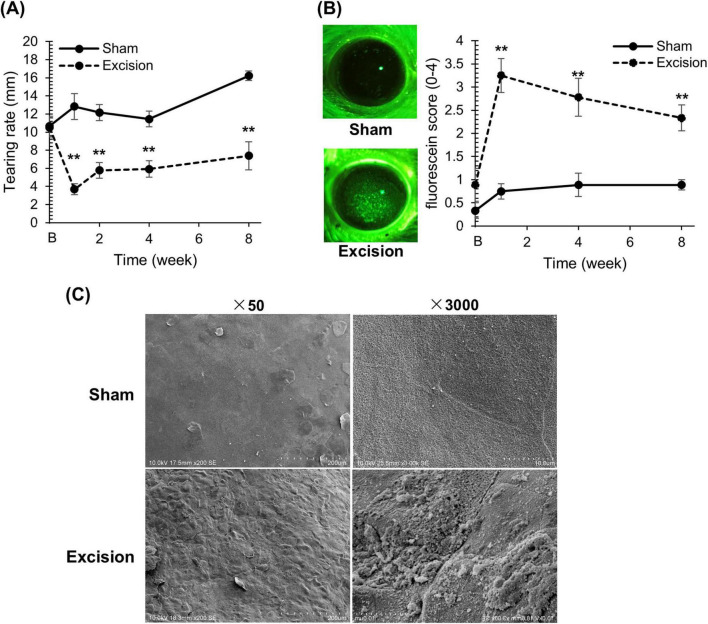
Changes in tear volume and epithelial damage on the cornea after lacrimal gland excision. **(A)** The length of the red-stained portion measured in the phenol red thread test between the sham versus excision sides. “B” in the transverse axis indicates the time point before surgery. Each point and the vertical bar represent the mean ± SEM (*n* = 9 for each group; Two-way ANOVA, **P < 0.01 compared to the sham side). **(B)** Left panel: Representative corneal images of fluorescein staining on the sham and excision sides. Right panel: Fluorescein staining score of the sham and excision sides. “B” in the transverse axis indicates the time point before surgery. Each point and the vertical bar represent the mean ± SEM (*n* = 9 for all groups, except for group 1 week where one sample was missing resulting in *n* = 8; Welch’s *t*-test or Mann–Whitney U test with Bonferroni correction, **P < 0.01 compared to sham side). **(C)** Scanning electron microscopy image of the corneal surface for the sham and excision sides. Left: Low-power field (original magnification, × 50; scale bar, 200 μm). Right: High-power field (original magnification, × 3,000; scale bar, 10 μm).

Before surgery and at 1, 4, and 8 weeks after surgery, we investigated the effects of both the extraorbital and intraorbital lacrimal gland excisions on the volume of tear fluid and corneal epithelial damage. The mean tear fluid volume, assessed using phenol red threads, significantly decreased on the excision side at all time points after surgery compared to that on the sham side (Figure 2A). Fluorescein staining was performed to evaluate corneal epithelial damage. The corneas on the excised side showed numerous punctate stains in the center, indicating epithelial damage. Gland excision significantly increased the fluorescein scores at all time points (Figure 2B).

To analyze the epithelial damage detected by fluorescein staining on the excision side in detail, we compared the corneal microstructure between the sham and excision sides 8 weeks after surgery using a scanning electron microscope. On the excision side, unevenness was extensively observed in the cornea compared to the sham side (× 50 magnification). Furthermore, while squamous epithelial cells on the sham side exhibited tight cell connections, those on the excised side showed impaired and loose connections (× 3,000 magnification) (Figure 2C).

### 3.2 Lacrimal gland excision induces dysesthesia on the ocular surface

**FIGURE 3:**
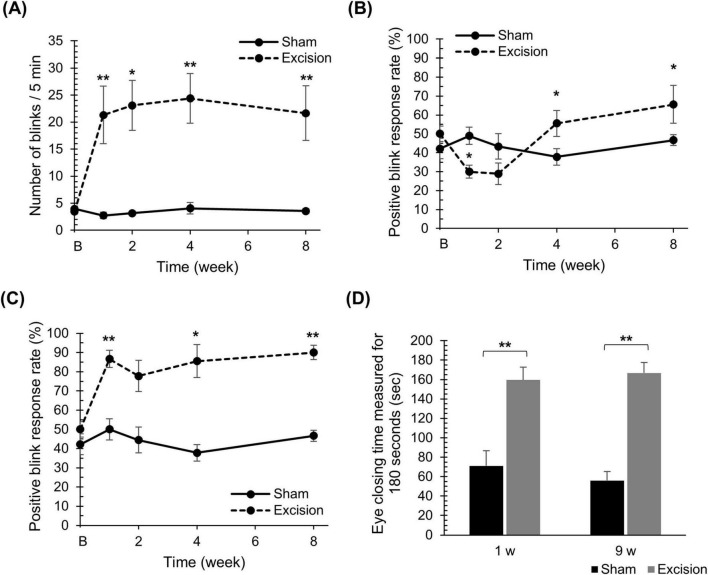
Changes in spontaneous blink behaviors, blink behaviors induced by mechanical stimulation, and eye-closure behaviors induced by chemical stimulation on the cornea after lacrimal gland excision. **(A)** The number of spontaneous blinks over 5 min for the sham compared to the excision sides. “B” in the transverse axis indicates the time point before surgery. Each point and the vertical bar represent the mean ± SEM (*n* = 9 for each group; Student’s *t*-test or Mann–Whitney U test with Bonferroni correction, *P < 0.05 and **P < 0.01 compared to the sham side). **(B)** The percentage of positive blink responses induced by mechanical stimulation with a force of 0.008 *g* upon pressing. “B” in the transverse axis indicates the time point before surgery. Each point and the vertical bar represent the mean ± SEM (*n* = 9 for each group; Two-way ANOVA, *P < 0.05 compared to the sham side). **(C)** The percentage of positive blink behaviors induced by mechanical stimulation with a force of 0.008 *g*, including blinks at the moment of release from the stimulus. “B” in the transverse axis indicates the time point before surgery. Each point and the vertical bar represent the mean ± SEM (*n* = 9 for each group; Student’s *t*-test or Mann–Whitney U test with Bonferroni correction, *P < 0.05 and **P < 0.01 compared to the sham side). **(D)** The total eye-closure time for 3 min induced by an eye drop with 100 μM capsaicin. Each column and the vertical bar represent the mean ± SEM (one week post-surgery; *n* = 5 for each group; Student’s *t*-test, 9 weeks post-surgery; *n* = 3 for each group; Student’s *t*-test, **P < 0.01 compared to the sham side).

To determine the ocular surface sensation in the present DED model, behavioral tests were performed using eye blinks or eye-closure behaviors during rest and in response to stimuli. The number of spontaneous blinks significantly increased at 1, 2, 4, and 8 weeks after surgery on the excision side compared with the sham side (Figure 3A). Next, we measured the mechanical sensitivity of the cornea using the positive blink response rate during 0.008 *g* stimulation by the von Frey filament. In the normal cornea, as shown on the sham side, the filament induced blink behaviors with probabilities of 40%–50% during the application of the force, whereas blink behaviors were rarely observed after releasing the force. On the excision side, the blink response rate during force application on the corneal surface significantly decreased at 1 week, and significantly increased at 4 and 8 weeks after surgery (Figure 3B). Interestingly, blinking behavior was frequently observed immediately after releasing the filament on the excision side, even when the blink was absent during stimulation, and the total positive blink response rate during and after mechanical stimulation significantly increased at 1, 4, and 8 weeks after surgery (Figure 3C). Finally, the sensitivities for nociceptive chemical stimulation were analyzed with an eye drop of 100 μM capsaicin, a TRPV1 agonist. The capsaicin-induced eye-closure time at both 1 and 9 weeks after surgery was significantly prolonged on the excision side than on the sham side (Figure 3D).

### 3.3 Lacrimal gland excision induces severe inflammation and drastically alters corneal nerve density

**FIGURE 4:**
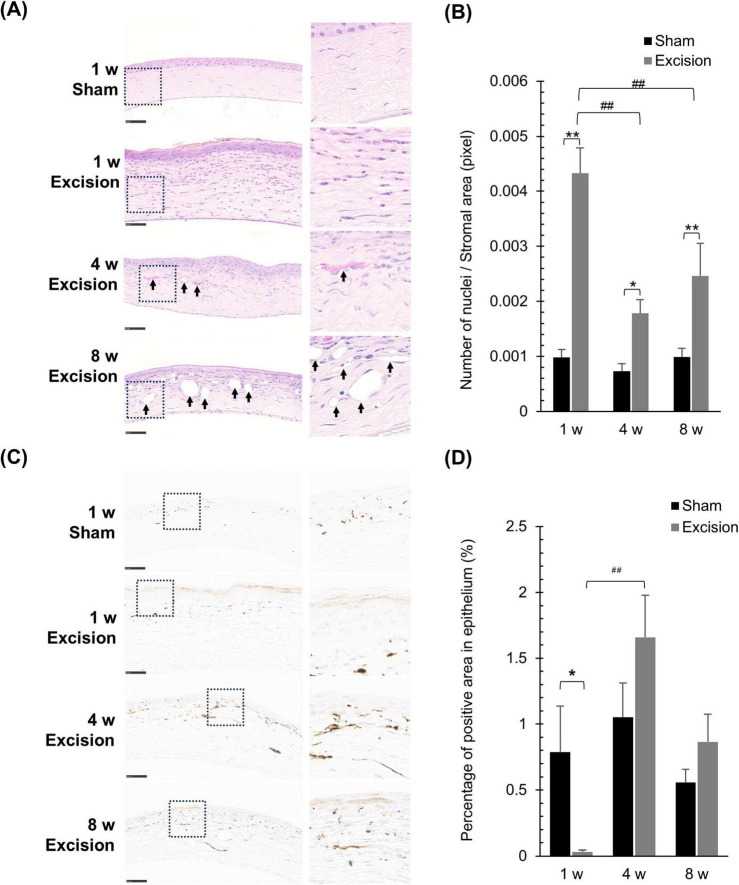
Histological alterations in the cornea at different time points after lacrimal gland excision. **(A)** Light microscopy images of the central cornea after hematoxylin and eosin staining for the sham side at 1 week post-surgery and the excision side at 1, 4 and 8 week post-surgery (Left panel: original magnification, × 40; scale bar, 50 μm. Right panel: magnified images within the dashed line in the left panel.). The arrow indicates the neovascular formations in the stroma. **(B)** Quantitative analysis of the number of cell nuclei in the stroma. Results are presented as the mean ± SEM (*n* = 5 for each group. Statistical significance was assessed using two-way ANOVA, *P < 0.05 and **P < 0.01, versus the sham side; ^##^*P* < 0.01 versus 1-week post-surgery). **(C)** Light microscopy images of the central cornea after tubulin βIII–immunostaining for the sham side at 1 week post-surgery and the excision side at 1, 4, and 8 weeks post-surgery (Left panel: original magnification, × 40; scale bar, 50 μm. Right panel: magnified images within the dashed line in the left panel.). **(D)** Quantitative analysis of the tubulin βIII-positive area in the epithelium, representing nerve density. Each column and the vertical bar represent the mean ± SEM (*n* = 5 for each group; Two-way ANOVA, *P < 0.05 compared to the sham side, and ^##^*P* < 0.01 compared to the 1-week post-surgery).

The time course of histological changes was investigated using sagittal corneal sections after excision of both the extraorbital and intraorbital lacrimal glands. On the excision side, various types of cells, including inflammatory and vascular cells, were observed in the corneas at all time points post-surgery than the sham side, particularly in the stromal layer. One week after surgery, epithelial and stromal hyperplasia along with acute inflammation characterized by the presence of inflammatory cells with segmented nuclei were observed. Subsequently, neovascularization in the stromal layer was noted at 4- and 8-weeks post-surgery, accompanied by chronic inflammation characterized by the infiltration of various inflammatory cells with round or irregular nuclear contours, in addition to segmented nuclei (Figure 4A). The number of cell nuclei in the stromal area increased at all time points after surgery, particularly at 1 week after surgery (Figure 4B). From 1 to 4 weeks, vascular structures increased in a time-dependent manner in the stroma of the central cornea on the excision side, although individual differences in vascularization were large. The number of vascular structures per stromal area increased at 4 and 8 weeks postoperatively in individuals with vascularized corneas (Table 1).

**TABLE 1:** Evaluation of neovascularization in the stroma of the central cornea at different time points following lacrimal glands excision.

Evaluation items	1 w		4 w		8 w	
	Sham	Excision	Sham	Excision	Sham	Excision
Number of individuals with vascular structures	0/5	2/5	0/5	4/5	0/5	3/5
Number of vascular structures per stromal area (pixels) in individuals with vascular structures	–	2.14 × 10^–5^	–	10.07 × 10^–5^ ± 3.04 × 10^–5^	–	23.87 × 10^–5^ ± 3.91 × 10^–5^

Next, to analyze the changes in the running condition of the nerve fibers, we visualized tubulin βIII immunostaining and compared the corneal nerves between the sham and excised sides. On the sham side, numerous nerve terminals and fibers were observed within the epithelium and stroma along with the epithelium, whereas only a few thick nerves were observed in the deep stromal portion (Figure 4C). One week postoperatively, nerve density within the epithelium, including the subbasal plexus, significantly decreased on the excision side, whereas thick nerve fibers markedly increased in the deep portion of the stroma. Subsequently, the epithelial nerve density recovered at 4 and 8 weeks after surgery (Figures 4C, D). Persistent excessive nerve fiber growth was observed in the deep stroma (Figure 4C).

### 3.4 The distribution of corneal CGRP-positive nerves is altered in the corneas after the lacrimal gland excision

**FIGURE 5:**
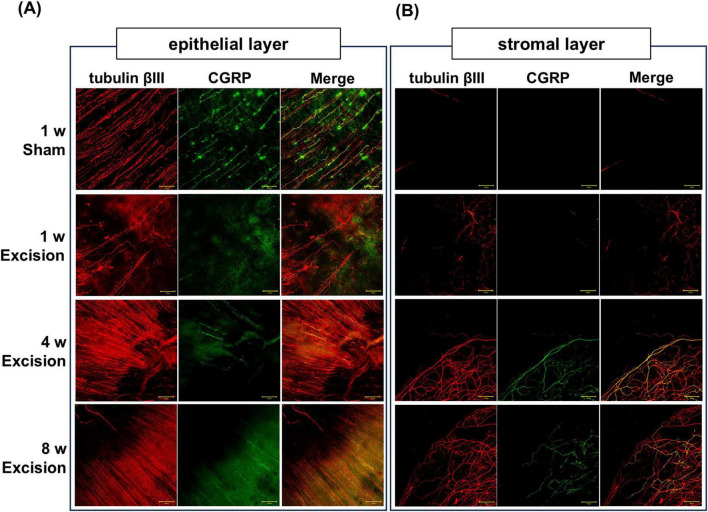
Changes in corneal nerve morphology in whole-mount preparations after lacrimal gland excision. The localization of tubulin βIII–positive nerve fibers (red) and CGRP–positive nerve fibers (green) in the sham side at 1 week post-surgery and the excision side at 1, 4, and 8 weeks post-surgery. **(A)** Corneal subbasal nerve plexus in the epithelium (original magnification, × 20; scale bar, 50 μm). **(B)** Corneal nerve innervation in the stroma at a depth of approximately 50–100 μm from the corneal surface (original magnification, × 20; scale bar, 50 μm).

CGRP is highly expressed in polymodal nociceptive nerve fibers (Belmonte et al., 2017). To reveal the morphological alteration of the nociceptive afferents, the distribution of tubulin βIII positive nerve fibers and CGRP-positive peptidergic nerves were analyzed using whole-mount immunostaining of the cornea. On the sham side, numerous nerve plexuses containing CGRP-positive fibers were observed extending from the limbus to the central cornea in the stroma neighboring the epithelium, whereas nerves were rarely observed in the stroma apart from the epithelium. One week after surgery, many nerve plexuses were denervated on the excision side, although a small number of nerve plexuses near the center of the cornea remained at depths near the epithelium. In addition, CGRP-positive fibers were almost absent in this area. Subsequently, the corneal nerve plexuses, including CGRP-positive fibers, were retrieved 4 and 8 weeks after surgery (Figure 5A). Additionally, from 1 week after surgery, thick nerve fibers containing CGRP-positive ones largely expanded into the stroma at a depth of approximately 50–100 μm from the surface (Figure 5B).

### 3.5 Expression of TRPV1 in the trigeminal ganglion remains unchanged 1 week after the lacrimal gland excisions

**FIGURE 6:**
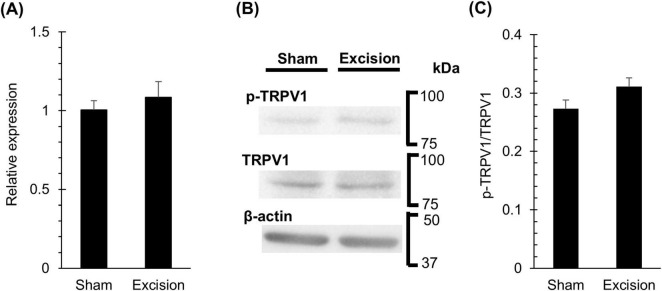
Changes in TRPV1 mRNA and protein in trigeminal ganglion one week after lacrimal gland excision. **(A)** The levels of TRPV1 mRNA normalizing to GAPDH in the trigeminal ganglion on the sham side versus the excision sides at 1-week post-surgery, measured using a qPCR method. Each column and the vertical bar represent the mean ± SEM (*n* = 5 for each group; Student’s *t*-test). **(B)** Representative western blot images of TRPV1, phosphorylated TRPV1, and β-actin bands in the trigeminal ganglion on the sham and excision sides at 1-week post-surgery. **(C)** The quantitative results comparing the ratio of total TRPV1 to phosphorylated TRPV1 protein in the trigeminal ganglion on the sham side versus the excision side. Each column and the vertical bar represent the mean ± SEM (*n* = 4 for each group; Student’s *t*-test).

The responsiveness to capsaicin eye drops increased one week after surgery, despite the denervation of CGRP-positive corneal nerves. Therefore, we investigated the changes in the mRNA and protein expression levels of TRPV1 in the trigeminal ganglion 1 week after surgery to examine whether the increase in capsaicin sensitivity on the ocular surface was accompanied by TRPV1 upregulation in the early phase of DED. The mRNA expression levels of TRPV1 measured by qPCR showed no significant difference between the sham and excised sides (Figure 6A). The ratio of total TRPV1 to phosphorylated TRPV1 protein, as measured by western blotting, also showed no significant difference between the two sides (Figures 6B, C).

### 3.6 The lacrimal gland excision alters the corneal localization of axon guidance factors

**FIGURE 7:**
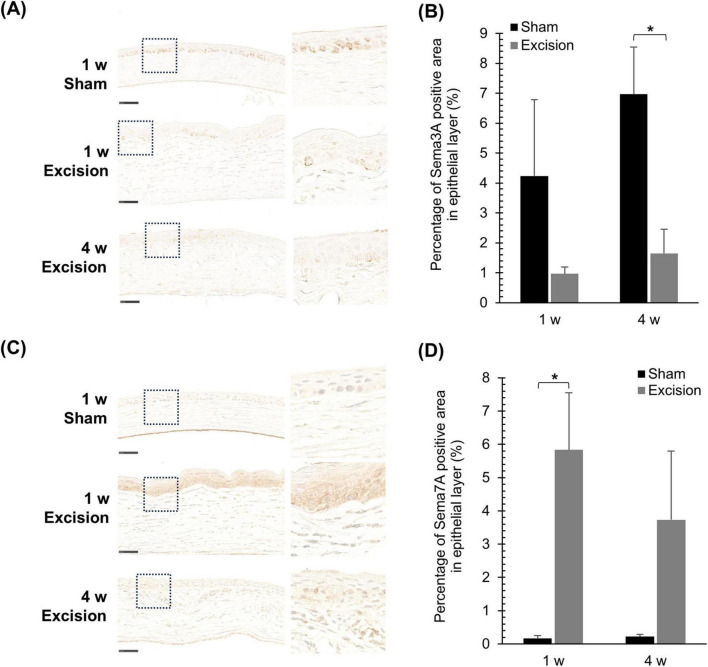
Changes in the localization of Sema3A and Sema7A expression after lacrimal gland excision. **(A)** Light microscopy images of the central cornea after Sema3A-immunostaining for the sham side at 1-week post-surgery and the excision side at 1 and 4 weeks post-surgery (Left panel: original magnification, × 40; scale bar, 50 μm. Right panel: magnified images within the dashed line in the left panel). **(B)** Quantitative analysis of Sema3A-positive area in the epithelium. Each column and the vertical bar represent the mean ± SEM (*n* = 5 for each group; Two-way ANOVA, *P < 0.05 compared to the sham side). **(C)** Light microscopy images of the central cornea after Sema7A immunostaining for the sham side at 1-week post-surgery and the excision side at 1 and 4 weeks post-surgery (Left panel: original magnification, × 40; scale bar, 50 μm. Right panel: magnified images within the dashed line in the left panel). (**D**) Quantitative analysis of Sema7A-positive area in the epithelium. Each column and the vertical bar represent the mean ± SEM (*n* = 5 for each group; Mann–Whitney U with Bonferroni correction, *P < 0.05 compared to the sham side).

To investigate the regulation of corneal nerve remodeling in this animal model, the localization of axonal guidance factors was examined. First, we stained for repulsive axon guidance factor Sema3A, which prevents neural formation. In the cornea on the sham side, from 1 to 4 weeks after surgery, Sema3A was localized in both the epithelium and stroma, and was highly expressed in the basal cells of the epithelium. Contrastingly, the intensity of Sema3A expression in the basal cells weakened on the excision side, although Sema3A continued to be expressed in both layers (Figure 7A). The area of strong Sema3A expression decreased within the epithelium of the central cornea on the excised side. A significant difference was observed 4 weeks after surgery compared with the sham side (Figure 7B).

Localization of the neurotrophic factor Sema7A, which attracts nerves, was also examined. Sema7A was localized and weakly expressed in the epithelium on the sham-operated side, whereas its expression was barely detectable in the stroma. In the cornea on the excision side, Sema7A was localized in both the epithelium and stroma, with markedly increased expression in the epithelium (Figure 7C). In the quantified data, the area highly expressing Sema7A in the epithelium significantly increased one week after surgery compared to that on the sham side (Figure 7D). Additionally, the fluorescent immunostaining images were also observed in order to confirm the Sema7A localization; the localization of Sema7A was similar to that described above (Suppplementry Figure 1A), though the corneal images showed autofluorescence and were unable to use for quantitative analysis (Suppplementry Figure 1B).

## 4 Discussion

In the present study, excision of the extraorbital and intraorbital lacrimal glands caused a persistent reduction in tear volume and remarkable corneal epithelial damage from 1 to 8 weeks post-surgery in male rats. Concurrently, the rats exhibited frequent blinking in the resting condition and aberrant eye-closure behavior due to mechanical and chemical stimuli, indicating the induction and facilitation of abnormal sensations on the ocular surface. In the images of the corneal pathological specimens, migration of inflammatory cells and neovascularization were observed. These observations suggest that these rats have characteristics of a severe form of DED, which is often caused by Sjögren’s syndrome (Chen et al., 2017).

In this DED rat model, the distribution of nerve fibers, including CGRP-positive nerves, was dramatically altered in the cornea. The nerve terminals and plexus were reduced in the first week after gland excision and subsequently regenerated in the correct direction, whereas the nerve bundles grew in the deeper stroma from an early period after excision, where few nerve bundles originally existed in the normal cornea. Previous animal studies have reported a decrease or no change in the epithelial corneal nerve density, regardless of the type of DED animal model (Fakih et al., 2019; Kovács et al., 2016). Sullivan et al. (2022) reported a similar result to our data; a significant reduction in tubulin βIII-positive corneal nerve innervation was observed one week after lacrimal gland excision in male mice. They also noted the presence of a disorganized regenerative process in the corneal axon terminals 4 weeks post-surgery (Sullivan et al., 2022). However, the growth of nerve bundles in the stroma of patients with DED has rarely been discussed in their reports. Clinical studies using IVCM have also yielded inconsistent results regarding whether corneal nerve plexus density decreases or increases in DED patients (Giannaccare et al., 2019; Labbé et al., 2013; Uchino et al., 2023), although the vast majority of studies have shown a decrease in the intraepithelial corneal nerve (ICN), including intraepithelial corneal basal nerves (ICBN) and intraepithelial corneal nerve terminals (ICNT) (Vereertbrugghen and Galletti, 2022). This contradiction in clinical reports may be caused by dynamic time-dependent changes in nerve morphology in the epithelium and stroma under tear-deficient conditions.

The distribution of CGRP-positive nerves, representing a subset of polymodal nociceptive nerves in the cornea, showed almost the same morphological alterations. Most notably, however, in the present study, nerve bundles containing CCRP-positive fibers in the stroma markedly increased after the migration of inflammatory cells, followed by angiogenesis. Some clinical and basic research suggests that CGRP may be released from primary afferents, thereby influencing ocular surface pathology (Belmonte et al., 2004). For example, CGRP release owing to nerve excitation promotes wound healing through inflammatory responses and angiogenesis during tissue repair (Toda et al., 2008; Zidan et al., 2024). Thus, an increase in CGRP-positive fibers within the deeper stroma may contribute to neovascularization in DED model rats. Besides, it is also known that CGRP is involved in nociceptor sensitization through inflammatory processes, such as macrophage recruitment (Caillaud et al., 2019; McKay et al., 2019). Therefore, reinnervation and growth of these CGRP-positive fibers at 4 weeks post-surgery may contribute to potential alterations in ocular surface sensation and chronic cell infiltration or proliferation in the cornea. The present study supports the hypothesis that neuronal CGRP regulates chronic inflammation and angiogenesis in DED corneas.

In the present study, we assessed the corneal sensitivity to mechanical stimulation using a von Frey filament. The ratio of positive blink responses during the application of force decreased one week after excision, when the nerve terminals and plexus decreased in the cornea. Thereafter, mechanical sensitivity is facilitated by the reconstruction of nerve fibers in the epithelium. Therefore, the perception of mechanical information on the ocular surface changed from hypoesthesia to hyperesthesia, synchronized with the denervation and reinnervation of the corneal nerves. However, Meng et al. (2015) demonstrated an increased sensitivity to mechanical stimulation of the corneal surface in the same DED model from 1 to 8 weeks post-surgery using a Cochet-Bonnet esthesiometer. This discrepancy may be due to the criteria of the positive blink behaviors induced by mechanical stimulation; in the detailed observation of blink behavior at 1-week post-surgery in our experiments, few blink behaviors were observed on the excision side while pressing the corneal surface by the filament. However, blinking behavior occurred with a high probability when the filament was kept away from the corneal surface after pressing. Thus, the response to direct mechanical force may be attenuated in the early phase on the excision side; nevertheless, the physiological mechanisms of blinking behavior after pressing are not yet understood. Thereafter, the corneal sensation completely transitioned to hyperesthesia, accompanied by reinnervation of the corneal nerves. Even in clinical studies, the corneal mechanical sensation measured by the response to air-puff stimulus was inconsistent in patients with DED, exhibiting hypoesthesia or hyperesthesia based on patients (Galor et al., 2021). In addition to DED, laser *in situ* keratomileusis (LASIK) also induces corneal nerve reinnervation following denervation (Lee et al., 2002). Sensory perception of mechanical stimuli is reported to progress from hypoesthesia to hyperesthesia and eventually returns to normal (Gallar et al., 2004). Thus, hyperesthesia in DED may be caused by morphological reconstruction of the GCRP-positive corneal nerves, followed by functional alterations in mechanoreceptors, possibly through chronic inflammation.

In contrast to mechanical sensitivity, spontaneous blinking and nociceptive responses to eye drops of the TRPV1 agonist capsaicin were persistently facilitated from the beginning after surgery despite the cornea being denervated. DED is often accompanied by inflammation and is reported to upregulate the concentrations of pro-inflammatory cytokines, including interleukin-1, interleukin-6, and tumor necrosis factor-α in the cornea (Barabino et al., 2012). TRPV1 activity in sensory nerves is well established to be enhanced by pro-inflammatory cytokines and local inflammatory signals, such as prostaglandins, nerve growth factors, and bradykinin, via either direct activation or phosphorylation (Petho and Reeh, 2012; Pinho-Ribeiro et al., 2017). We have previously demonstrated the sensitization of TRPV1-mediated responses in the corneal nerves of guinea pigs with DED induced by lacrimal gland excision, referred to as corneal allodynia (Masuoka et al., 2020b). Elevated TRPV1 expression in cold-sensitive sensory nerves that express TRPM8 may enhance ocular surface pain in DED (Hatta et al., 2019; Li et al., 2019). The expression of TRPV1 protein in corneal neurons located in the trigeminal ganglion has been reported to be upregulated in DED model mice, induced by both extraorbital and intraorbital lacrimal gland excision 14 days after surgery (Sullivan et al., 2022). The mRNA of TRPV1 in the trigeminal ganglion was also increased in DED model mice exposed to low humidity for 14 days (Naderi et al., 2025). However, in our experiments, in the early stages of our DED model at 1-week post-surgery, no significant changes were observed in the gene and protein expression of TRPV1 or in the presence of phosphorylated TRPV1 in the trigeminal ganglion. Thus, potentiation of TRPV1-mediated eye-closure behavior in the early phase of chronic ocular dryness could be “inflammatory hyperalgesia” caused by local inflammation, though the influence of change in the efficiency of capsaicin to the polymodal nociceptor caused by epithelial damages is unable to be excluded.

The mechanisms underlying reinnervation of the corneal nerves in DED have not been sufficiently elucidated. The present study showed that the expression of Sema3A decreased, whereas that of Sema7A increased in the epithelium. Semaphorins are major axon-guidance factors that play crucial roles in nerve regeneration in various tissues (Caillaud et al., 2019). The repulsive axon guidance factor Sema3A, which is categorized as an extracellular matrix molecule, is localized in both the epithelium and stroma of adult rat corneas (Morishige et al., 2008). Several studies have suggested that the inhibitions of Sema3A signaling are suggested to promote corneal nerve regeneration after corneal injury. Omoto et al. (2012) reported that the Sema3A inhibitor SM-345431, isolated from the culture broth of *Penicillium* sp. SPF-3059 accelerates peripheral nerve regeneration and restores ocular surface sensation in a corneal transplantation model. Similarly, Yamazaki et al. (2017), reported that SM-345431 preserved corneal nerve and epithelial integrity in a mouse DED model. On the other hand, Sema7A is a glycosylphosphatidylinositol (GPI)-anchored membrane-associated semaphorin, and promotes axonal outgrowth through the activation of integrin receptors and MAPK signaling pathway (Okuno et al., 2011; Pasterkamp et al., 2003). Thus, the reduction in Sema3A and elevation of Sema7A in the epithelium are considered to promote the reinnervation of corneal nerves on the corneal surface. Additionally, Sema7A was expressed in infiltrating cells within the stroma of the DED cornea in the present study. Sema7A was reported to express in activated T cells and plays a crucial role in the effector phase of the inflammatory immune response by stimulating cytokine production in monocytes and macrophages (Suzuki et al., 2007). Therefore, infiltrating cells expressing Sema7A attract CGRP-positive nerves to the stroma, resulting in abnormal innervation and persistent inflammation.

Our findings suggest that hypoesthesia, followed by hyperesthesia to mechanical stimulation, coincides with changes in the corneal nerve density in the epithelium during the progression of severe DED. In contrast, the TRPV1-mediated pain response was consistently facilitated. The severity of the uncomfortable sensations experienced by patients with DED does not necessarily correlate with the corneal nerve density in the epithelium or with tactile perception. These findings suggest that evaluating painful sensations and mechanical sensitivity with a Cochet–Bonnet esthesiometer or corneal nerve density with confocal microscopy is valuable for identifying disease progression. Furthermore, the severity of corneal pathology, as indicated by inflammation and neovascularization, can be assessed through observing the localization of stromal nerve fibers.

Finally, the use of male rats and the contralateral eye as the control in unilateral lacrimal gland excision rats should be considered as methodological limitations when interpreting the results in this study. Ocular surface inflammation in unilateral eye functionally affects corneal sensory nerve activity in both the same-side and contralateral eyes (Luna et al., 2021). Additionally, sex and hormones affect the onset of DED, and DED more frequently occurs in women than in men (Sullivan et al., 2017).

## 5 Conclusion

In this study, the cornea under chronic ocular dryness was denervated and subsequently re-innervated within the epithelium and abnormally innervated in the stroma. Corneal CGRP-positive fibers exhibited similar structural changes. Abnormal sensitivity to mechanical stimulation was synchronized with the denervation and reinnervation of the corneal nerve plexuses in the epithelium. In contrast, the persistent hyperalgesia in response to capsaicin did not correlate with a reduced density of CGRP-positive nerves or the expression and phosphorylation of TRPV1 in the trigeminal ganglion during the early phase. However, excessive stromal innervation by CGRP-positive nerves may contribute to chronic corneal inflammation and angiogenesis. Finally, variations in the semaphorin proteins Sema3A and Sema7A were presumed to be involved in the reinnervation of DED as part of the regulatory mechanism.

## Data Availability

The original contributions presented in the study are included in this article/Supplementary material, further inquiries can be directed to the corresponding author(s).
