# Supplementary material for: Tear deficiency transforms spatial distribution of corneal calcitonin gene-related peptide-positive nerves in rats
Source: Front Cell Neurosci. 2025 Jul 1;19:1619310. doi: 10.3389/fncel.2025.1619310 (PMC12259659; doi:10.3389/fncel.2025.1619310)
Supplement: Supplementary file 1 [file Data_Sheet_1.pdf]

Supplementary Figures

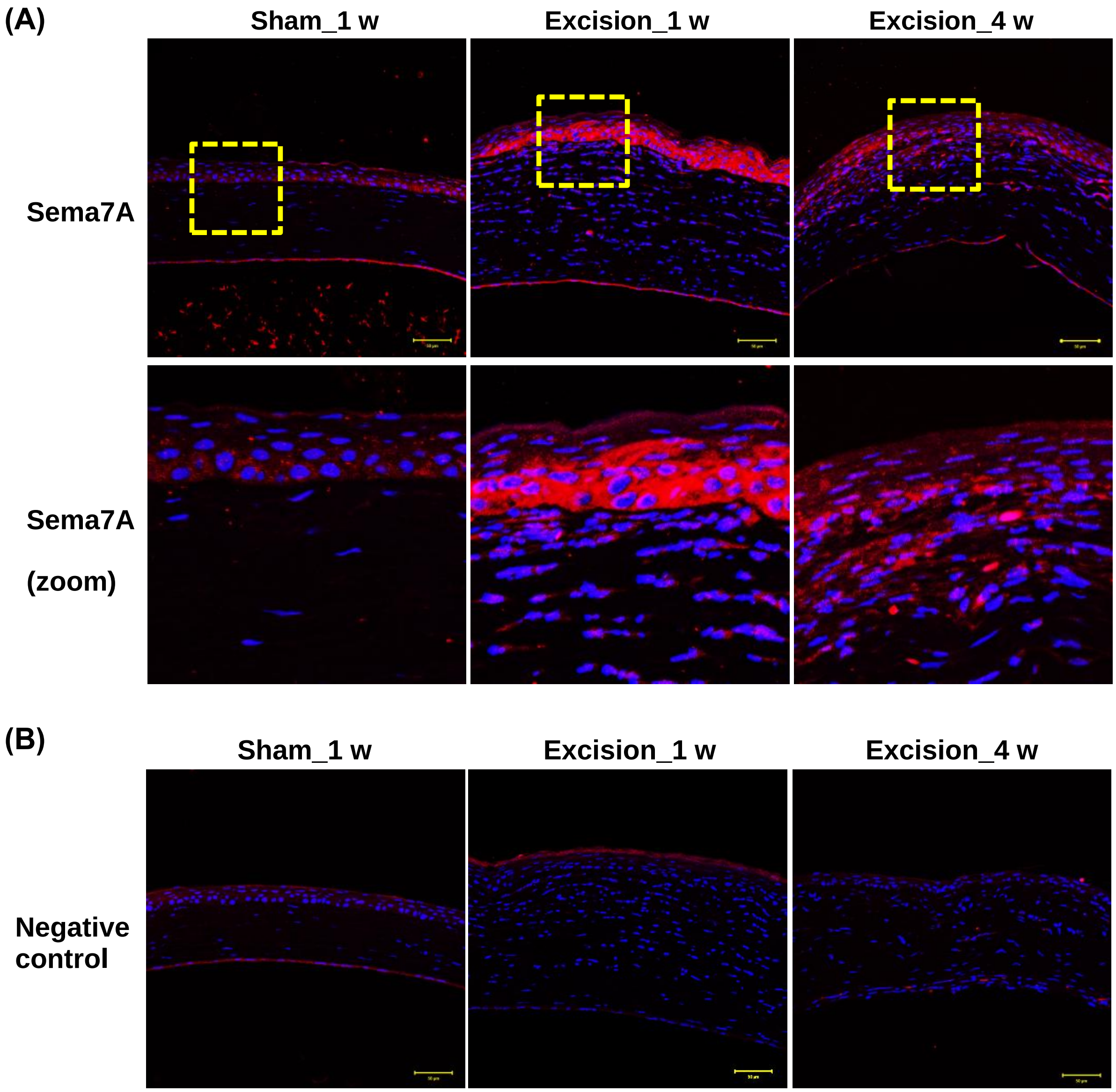

Supplemental figure 1. Immunofluorescent staining of Sema7A in the central cornea. (**A**) Upper images: Fluorescence images of the central corneal tissue following Sema7A-immunostaining in the sham side at 1-week post-surgery and the excision side at 1 and 4 weeks post-surgery (original magnification, ×20; scale bar, 50 µm). Lower images: Enlarged views of the area indicated by the yellow square in the upper images. (**B**) Autofluorescence images of the central corneal tissue serving as a negative control, where no primary antibody was applied, in the sham side at 1-week post-surgery and excision sides at 1 and 4 weeks post-surgery (original magnification ×20; scale bar: 50 µm).
